# Portal-Mesenteric Suppurative Emphysematous Pylephlebitis: A Case Report

**DOI:** 10.7759/cureus.41693

**Published:** 2023-07-11

**Authors:** Rida Cheikh Youssef, Jean-Marie Jacques, Soheil Zahir, Thierry Roger, Serge Landen

**Affiliations:** 1 Department of Emergency Medicine and Critical Care Unit, Delta Chirec Hospital, Brussels, BEL; 2 Department of Emergency Medicine, Epicura Hospital, Hornu, BEL; 3 Department of Emergency Medicine, Centre Hospitalier Régional Sambre et Meuse, Sambreville, BEL; 4 Department of Radiology, Delta Chirec Hospital, Brussels, BEL; 5 Department of Surgery, Delta Chirec Hospital, Brussels, BEL

**Keywords:** inferior mesenteric vein thrombosis (imvt), pylephlebitis, septic portal vein thrombosis, complicated diverticulitis, intra-abdominal infection

## Abstract

Suppurative thrombophlebitis of the portal-mesenteric venous system occurring in the setting of abdominal inflammatory and infectious processes is a serious condition that can lead to septic shock, bowel ischemia, hepatic abscess, and death if unrecognized. Diagnosis is often delayed because symptoms are aspecific and pain at the primary site of infection may be mild. Contrast-enhanced CT scans can diagnose both portal thrombosis and a primary infection site. Treatment may include early resective surgery in case of appendicitis or diverticulitis, in association with large-spectrum antibiotics and possibly anticoagulation. A characteristic of suppurative thrombophlebitis, whether splanchnic or systemic, is the latency before the effects of antibiotic therapy are seen. Anticoagulation can be administered to avoid extension to the superior mesenteric vein. We presented a critically ill 53-year-old man with chronic colonic diverticulitis complicated by suppurative emphysematous portal-mesenteric thrombophlebitis with only a slow response to large-spectrum antibiotics.

## Introduction

Suppurative thrombophlebitis of the portal system is a rare disease that can complicate various splanchnic infections and inflammations [1]. Its incidence is estimated at 2.7 cases per 100,000 persons per year [2]. Despite diagnostic and therapeutic progress, a delay in diagnosis and management is still responsible for an 8.7% mortality rate [3]. An autopsy study published by Waller in 1846 first described this condition as the source of liver abscess [4].

This condition can be difficult to diagnose due to its non-specific symptoms, including abdominal pain, nausea, vomiting, fever, asthenia, and jaundice in the case of liver involvement [1]. Because of the nonspecific nature of the symptoms, diagnosis is often delayed [5]. There is no consensus on the management of this condition as the medical literature is mostly limited to clinical cases or short series.

The patient described herein presented with septic shock of undetermined origin. Despite a history of diverticular abscess two years previously, there was a discrepancy between seemingly uncomplicated sigmoid diverticular disease on CT scan and the severity of sepsis. Our report demonstrates that repercussions of suppurative pylephlebitis can occult the symptoms due to the primary site of infection.

## Case presentation

A 53-year-old man was admitted to the emergency department with abdominal pain that had been present for two weeks, accompanied by fever, nausea, vomiting, and loss of appetite. His history included diverticular peritonitis of the left colon two years ago treated by laparoscopic lavage drainage, active smoking, and alcohol consumption. On admission, the heart rate was 120 beats per minute, mean arterial pressure 80 mmHg, respiratory rate 20 breaths per minute, pulsed oxygen saturation 99% on room air, and the temperature was 39.4 °C. Cardiopulmonary auscultation was unremarkable. The abdomen was diffusely tender with a rebound in the left iliac fossa. Peristalsis was present. Laboratory investigations and arterial blood gas were performed on admission. Autoimmune serology was normal. The relevant biological parameters are listed in Table 1. 

**Table 1:** Biological parameters. pH, potential of hydrogen; INR, international normalized ratio

Laboratory parameters	Results	Normal range
Haemoglobin	10.4	11.7-16.1 g/dL
White blood cells	8,100	4,500-10,000/mm³
Platelets	39,000	150,000-400,000/mm³
C-reactive protein	310	<5 mg/L
Aspartate aminotransferase	89	<32 U/L
Alanine aminotransferase	75	<33 U/L
Alkaline phosphatase	195	35-105 U/L
Gamma-glutamyl transferase	200	<36 U/L
Total bilirubin	4.5	<1.1 mg/dL
Direct bilirubin	4	<0.3 mg/dL
International Normalized Ratio (INR)	1.5	<1.5
Fibrinogen	822	200–393 mg/dL
Potentiel hydrogen (pH)	7.53	7.35-7.45
Partial pressure of carbon dioxide	39	35-45 mmHg
Partial pressure of oxygen	69	83-108 mmHg
Lactate	1,5	<1.3 mmol/L
Bicarbonate	31	21-28 mmol/L

**Figure 1:**
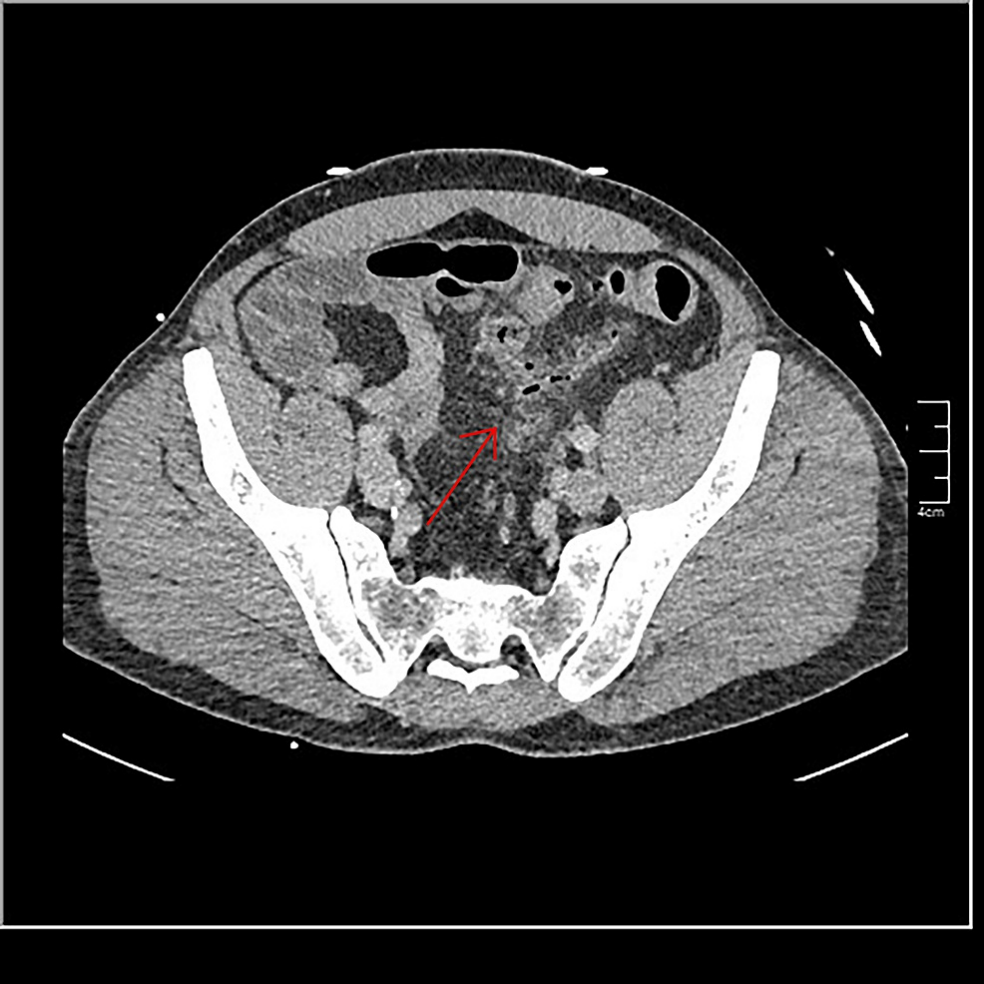
Abdominal CT scan with an injection of contrast showing thickening of the sigmoid colon with infiltration of the surrounding fat. CT, computed tomography

**Figure 2:**
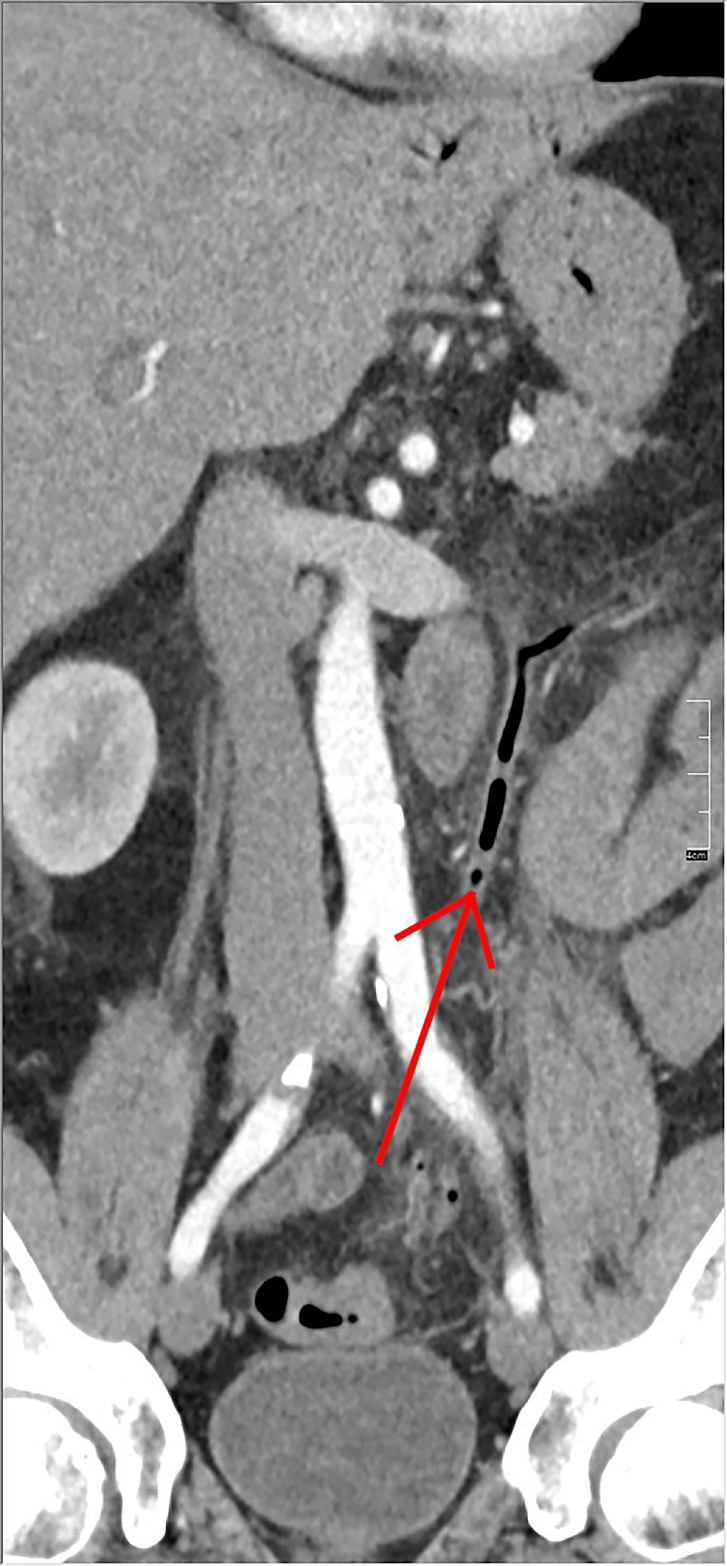
Abdominal CT scan with an injection of contrast showing thrombophlebitis and air within the inferior mesenteric vein. CT, computed tomography

**Figure 3:**
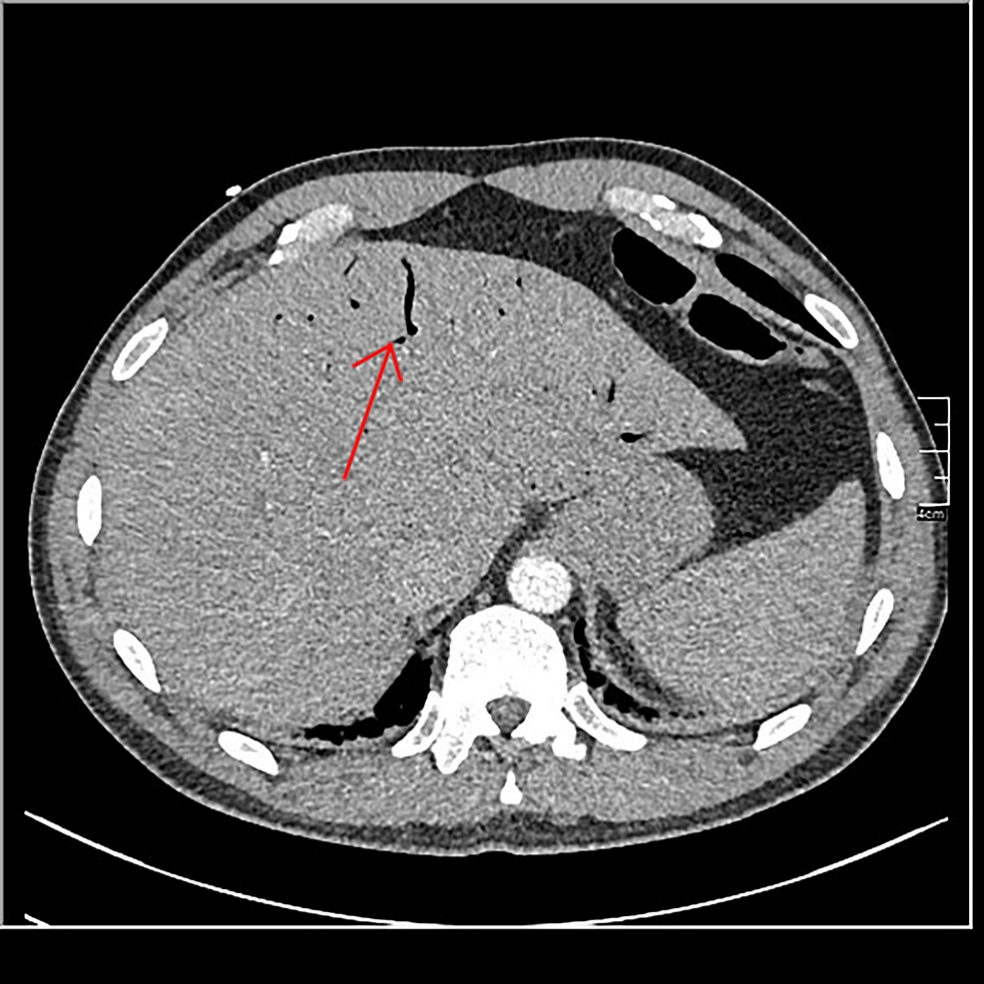
Abdominal CT scan of the liver showing air within the portal radicles. CT, computed tomography

**Figure 4:**
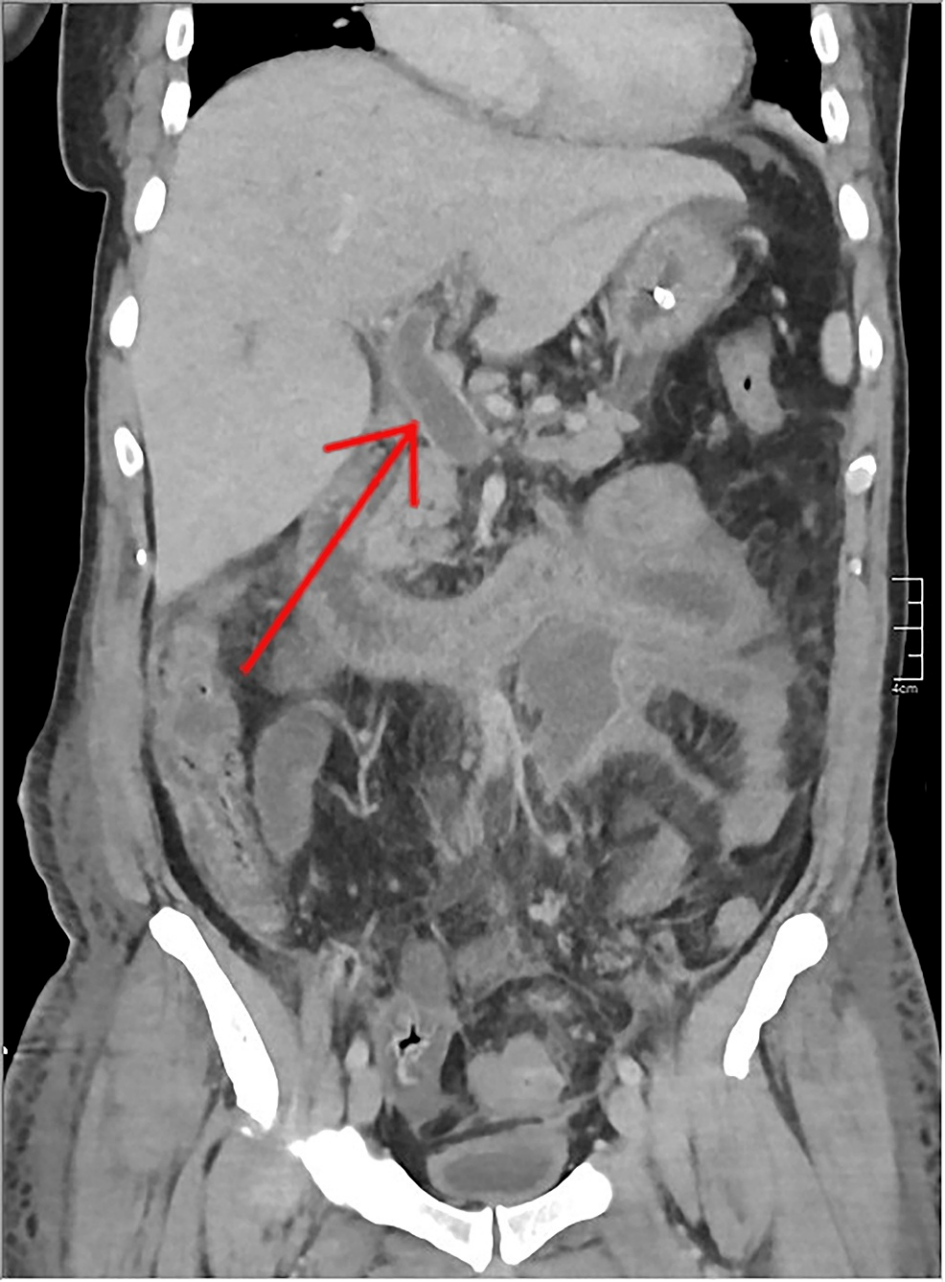
Abdominal CT scan in the portal phase showing portal vein thrombosis. CT, computed tomography

Abdominal CT scan with contrast showed thickening of the sigmoid colon with infiltration of surrounding fat (Figure 1). Thrombosis of the inferior mesenteric vein with aerial densities within it was noted (Figure 2). Sections on the liver reveal air within the portal radicles (Figure 3) and portal thrombosis ( Figure 4). There were no signs of peritonitis or digestive ischemia.

The patient was admitted to the critical care unit for management and monitoring. Antibiotic therapy based on amoxicillin-clavulanate was initiated (3 × 2 g/day). Several blood cultures showed the presence of clavulanate-resistant Escherichia coli (extended-spectrum beta-lactamases). The antibiotic therapy was changed to meropenem (3 × 1 g/day). Anticoagulation with unfractionated heparin was started immediately, followed by low-molecular-weight heparin. 

Given the severity of the sepsis and the presence of portal gas, exploratory laparoscopy was performed, which did not show peritonitis or intestinal ischemia. There were at least two dense adhesions between the sigmoid colon and the small bowel that were taken down. The procedure did not require conversion to open laparotomy. 

On postoperative day 7, the patient deteriorated hemodynamically due to worsening sepsis. Inotropic support by noradrenaline was administered, as well as intensification of antibiotic therapy with the addition of amikacin, ciprofloxacin, and fluconazole. On the respiratory side, a septic acute respiratory distress syndrome (ARDS) developed, justifying orotracheal intubation and invasive mechanical ventilation. The patient was reoperated by open laparotomy for exploration of the portal-mesenteric axis. Although the splanchnic veins were turgescent, there were no signs of bowel ischemia. Furthermore, there were no signs of overt complicated sigmoid diverticulitis. Due to the patient's critical condition, the presence of portal hypertension, and thrombocytopenia caused by sepsis, it was determined that sigmoidectomy would be postponed until a later stage. It was also felt that less extensive surgery would allow early restoration of heparin anticoagulation, which was considered important to avoid the extension of thrombosis to the superior mesenteric vein.

A cholecystectomy was performed to improve access to the hepatic pedicle. A phlebotomy of the portal vein and the superior mesenteric vein was performed followed by the passage of a Fogarty probe. However, it was not possible to recanalize the venous axis, whose lumen was occupied by an organized thrombosis. The bacteriological analysis of the thrombosis was negative.

Histological analysis of the gall bladder showed chronic cholecystitis with no signs of malignancy. 

The postoperative evolution was slowly favorable, and the patient was discharged from intensive care on the 21st day. Total parenteral nutrition was initiated. Pre-heparin therapy coagulation tests were normal, revealing no prothrombotic state. Anticoagulation was continued for six months. 

**Figure 5:**
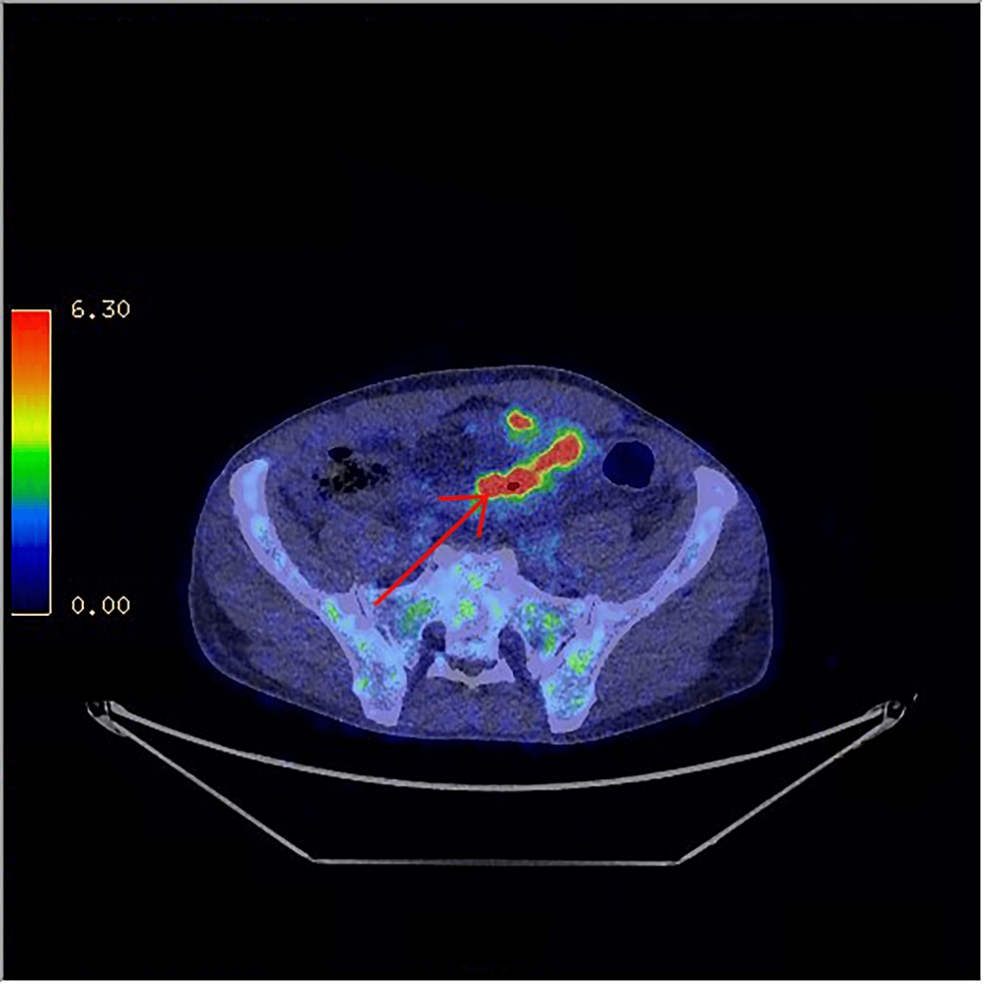
PET CT scan showing a persistent hypermetabolic focus in the sigmoid colon after one month of discharge from the hospital. PET, positron emission tomography; CT, computed tomography

One month after his discharge from the hospital, he had intermittent febrile spikes. Blood tests were normal, and new blood cultures were negative. The PET scan showed a persistent hypermetabolic focus in the sigmoid colon (Figure 5). A total ileocolonoscopy showed only a short segment with chronic sequelae of sigmoid diverticulitis and no evidence of Crohn's disease.

At the two-year follow-up, the patient recovered fully but developed a portal cavernoma and grade 1 esophageal varices. He, however, declined sigmoidectomy.

## Discussion

The etiology of suppurative thrombophlebitis of the portal-mesenteric axis includes diverticulitis (30%), appendicitis (9%), chronic inflammatory bowel disease (6%), acute pancreatitis (5%), infectious enteritis (4%), bowel perforation, and bowel neoplasia (6%) [6-9].

Suppurative thrombophlebitis results from an infectious process originating in the organs drained by the portal system. It involves the peripheral veins and then extends to the portal vein and intrahepatic portal branches. Upper portal-mesenteric thrombosis can cause intestinal ischemia and peritonitis. Other complications include sepsis, septic shock, and liver abscesses [10].

In children, it results mainly from neonatal umbilical infection or septic cannulation of the umbilical vein. In a series of 95 patients, the largest reported to date by Choudhry et al., suppurative thrombophlebitis affected adults of all ages and the majority had diverticulitis or appendicitis [11].

Congenital or acquired coagulation disorders are contributing factors [12]. Rarely has suppurative pylephlebitis been described after hemorrhoidal ligation, liver biopsy, or gastric banding [13-15].

More rarely, this condition may complicate the course of chronic inflammatory bowel disease [7,8]. In 1946, Taylor described for the first time suppurative thrombophlebitis complicating Crohn's disease [16]. Currently, there are only sporadic cases in the literature of suppurative thrombophlebitis in patients with Crohn's disease [7,17]. Although rare in Crohn's disease, suppurative thrombophlebitis of the portal vein often reflects advanced disease. Scaringi et al. reported on a patient with fistulizing Crohn's disease complicated by suppurative mesenteric and portal thrombophlebitis [18].

Signs and symptoms are nonspecific, including fatigue, spikes of fever, abdominal pain, nausea, vomiting, diarrhea, and anorexia [1,9]. The most advanced cases present hepatomegaly, cholestasis, and hepatic cytolysis, indicating septic damage to the liver. From then on, the clinician focuses on the hepatic damage and may first hypothesize angiocholitis.

Leucocytosis is often elevated early on, but normocytosis and even leukopenia are possible. Bacteremia and elevated temperature in a patient with chronic splanchnic inflammation without acute painful exacerbation should raise the possibility of concomitant suppurative thrombophlebitis. Pylephlebitis can be diagnosed via abdominal ultrasonography. An abdominal CT scan with intravenous contrast injection is more widely used because of its ability to detect other sources of infection in the abdomen.

Baril et al. showed that the site of thrombosis was mainly in the portal vein. More rarely, the infection was limited to the inferior mesenteric vein [9].

In contrast to the blood culture, the culture of the portal thrombosis of our patient did not reveal the presence of germs. The infection is usually polymicrobial [1,6]. Choudhry et al. showed that only 44% of patients had a germ identified by thrombus culture [11]. In their series of 95 cases, the most common germ involved was Streptococcus viridans, E. coli, Bacteroides fragilis, and Streptococcus anginosus [11]. Kanellopoulou et al. also found S. viridans in their patients [19].

Several studies recommended broad-spectrum antibiotic therapy, even in the absence of bacteremia [6,9,11]. A characteristic of suppurative thrombophlebitis, whether splanchnic or systemic, is the latency before the effects of antibiotic therapy are seen. The ideal duration of antibiotic therapy for pylephlebitis is unclear. Plemmons et al. suggested between four and six weeks of antibiotherapy [6].

The use of anticoagulation remains controversial, with experience limited to isolated cases. There are no studies that formally demonstrate the usefulness of anticoagulation. The primary aim of anticoagulation is to prevent the extension of the thrombosis to the superior mesenteric venous axis. The secondary objective is venous repermeabilization, which is less unlikely, contrasting with its efficacy in aseptic thrombosis. Plemmons et al. noted a 100% survival in anticoagulated patients compared to 60% in patients who did not receive anticoagulation [6].

Allaix et al. considered that six months of anticoagulation treatment was appropriate if no prothrombotic disorders were identified [20]. There have been anecdotal reports of systemic thrombolysis, local thrombolysis, and percutaneous thrombectomy, but their usefulness has not been demonstrated.

## Conclusions

Suppurative thrombophlebitis of the mesenteric-portal axis is a rare and potentially fatal condition. It is most often concomitant with acute digestive infections and inflammations such as Crohn's disease. It is best diagnosed by a CT scan with an injection of contrast medium in the portal phase. Antibiotic therapy and possibly anticoagulation are the key elements in its management.
